# Rational Discovery of Antiviral Whey Protein-Derived Small Peptides Targeting the SARS-CoV-2 Main Protease

**DOI:** 10.3390/biomedicines10051067

**Published:** 2022-05-04

**Authors:** Nicola Gambacorta, Leonardo Caputo, Laura Quintieri, Linda Monaci, Fulvio Ciriaco, Orazio Nicolotti

**Affiliations:** 1 Dipartimento di Farmacia Scienze del Farmaco, Università degli Studi di Bari “Aldo Moro”, Via E. Orabona, 4, I-70125 Bari, Italy; nicola.gambacorta1@uniba.it; 2 Institute of Sciences of Food Production, National Research Council of Italy, 70126 Bari, Italy; leonardo.caputo@ispa.cnr.it (L.C.); linda.monaci@ispa.cnr.it (L.M.); 3 Dipartimento di Chimica, Università degli Studi di Bari “Aldo Moro”, Via E. Orabona, 4, I-70125 Bari, Italy; fulvio.ciriaco@uniba.it

**Keywords:** antiviral peptides, protease inhibitors, molecular docking, rhinovirus, COVID-19, milk

## Abstract

In the present work, and for the first time, three whey protein-derived peptides (IAEK, IPAVF, MHI), endowed with ACE inhibitory activity, were examined for their antiviral activity against the SARS-CoV-2 3C-like protease (3CL^pro^) and Human Rhinovirus 3C protease (3C^pro^) by employing molecular docking. Computational studies showed reliable binding poses within 3CL^pro^ for the three investigated small peptides, considering docking scores as well as the binding free energy values. Validation by in vitro experiments confirmed these results. In particular, IPAVF exhibited the highest inhibitory activity by returning an IC_50_ equal to 1.21 μM; it was followed by IAEK, which registered an IC_50_ of 154.40 μM, whereas MHI was less active with an IC_50_ equal to 2700.62 μM. On the other hand, none of the assayed peptides registered inhibitory activity against 3C^pro^. Based on these results, the herein presented small peptides are introduced as promising molecules to be exploited in the development of “target-specific antiviral” agents against SARS-CoV-2.

## 1. Introduction

On 11 March 2020, the World Health Organization (WHO) declared the novel coronavirus (COVID-19) outbreak a global pandemic for the rapid spread of severe acute respiratory syndrome coronavirus-2 (SARS-CoV-2) worldwide [1]. To date, the SARS-CoV-2 infection has caused more than 4 million deaths around the world [2].

The advent of effective vaccines and the adoption of restrictive prophylaxis measures, as well as appropriate sanitization procedures, have improved the global fight against SARS-CoV-2 [3]. However, the world still urgently needs new and affordable approaches to better counteract the spread of SARS-CoV-2 and prevent the spectrum of lethal adverse effects, especially in immunocompromised and vulnerable people. Thus, a wide range of therapies tackling the effects of COVID-19 in frail, symptomatic patients is increasingly gaining ground in clinical practice. They include both antivirals based on nucleotide analogs, such as Paxlovid™ (Nirmatrelvir and Ritonavir, Pfizer), Malnupirovir™ (Merck), Favipiravir, Remdesivir, and corticosteroids (such as Dexamethasone), and cytokine inhibitors (baricitinib and anti-IL-antibodies 6, anakinra). In addition, the use of monoclonal antibodies as an alternative to convalescent plasma is also spreading. Antivirals have been developed to block viral replication by inhibiting the RNA-dependent RNA replicase. Conversely, corticosteroids are used to avoid severe forms of the disease [4,5]. However, these drugs must be taken while under medical supervision or even in a clinical environment and they can often show serious adverse side effects. Furthermore, they all have a very high cost, preventing their large-scale diffusion [4,6]. Thus, the discovery of new molecular entities, showing low toxicity and high specificity, to block the entry or replication of SARS-CoV-2 in host cells still represents a challenge regarding safer and more effective therapeutic solutions [7].

The SARS-CoV-2 3-chymotrypsin-like cysteine protease (3CL^pro^), also named the main protease (M^pro^), which plays an important role in the virus’s life cycle, is considered an attractive target for the discovery of promising antiviral agents [8]. Importantly, 3CL^pro^ is involved in the replication process due to its two N-terminal domains, containing two β-barrel chymotrypsin-like folds ultimately responsible for the cleavage of the viral polyproteins to yield 16 mature      non-structural proteins [9,10].

Human rhinoviruses (HRV, belonging to the Picornaviridae family), etiological agents of the common cold, also show a protease (HRV 3C protease or 3C^pro^) involved in the viral replication process. Briefly, 3C^pro^ and 3CL^pro^ are cysteine proteases and share a typical chymotrypsin-like folding, a nucleophilic cysteine residue in the active site, and a preference for a glutamine or glutamic acid residue in the primary binding residue (P1 site) of the substrate proteins. Picornaviral HRV 3C protease was studied in the recent past for its degree of homology with coronaviral 3C-like proteases (3CL^pro^) within the 3C coding region, including the strict conservation of the active-site residues, thus providing an additional rationale for targeting drug discovery efforts [11,12]. Nevertheless, subtle differences in the active-site structures of these proteases did not allow for the identification of common inhibitors [13]. Only recently, a duplex assay based on self-assembled monolayer desorption ionization (SAMDI)-MS analysis has been developed, allowing the identification of equipotent peptides against 3CL^pro^ and HRV 3C^pro^ [14].

Several studies have been carried out regarding the structural and functional characterization of these proteins, to be employed for high-throughput screening for the discovery of new effective inhibitors [15,16,17]. The search for 3CL^pro^ inhibitors has been pursued by exploiting FDA-approved drugs, screening the libraries of natural and chemical compounds, and considering the de novo design of novel agents [18,19,20]. Based on these strategies, a number of putative inhibitors, whose structures can be grouped as peptidic and non-peptidic, have been identified and are awaiting further investigations before approval [16,21,22,23]. Recently, the 3CL^pro^ covalent inhibitor Nirmatrelvir (PF-07321332, purchased in combination with Ritonavir) has been authorized for emergency use for COVID-19 patients not requiring supplemental oxygen [24,25].

In order to increase the affinity and efficacy, 3CL^pro^ inhibitor substrates have been mainly modified by introducing some reactive chemical groups, such as Michael acceptors, aldehydes, epoxy ketones, and so on [8]. However, these structural changes promote the formation of covalent bonds with the catalytic cysteine responsible for the enzyme’s irreversible inhibition and, thus, for potential toxicity [14,16].

Besides synthetic compounds, other studies on promising antiviral agents are focusing on the identification of bioactive molecules from natural sources [26,27,28]. Among others, health-promoting - represent an attractive option. It has been reported that the role of food ingredients and active components (i.e., bioactive peptides, polysaccharides, bioactive lipids, and natural polyphenols) in supporting immune function in the prevention and treatment of COVID-19 disease is important [29,30]. Noteworthy, several peptides from milk proteins showed their ability to inhibit the main SARS-CoV-2 proteases [30,31]. Natural peptides show lower toxicity and fewer side effects: their application in adjuvant therapies is also favored by their satisfactory biological activity profiles, in which antioxidant, antimicrobial, immunomodulatory, anti-inflammatory, and/or angiotensin-converting enzyme (ACE) inhibitory activities can coexist [26,32,33]. Interestingly, peptides taken from milk proteins and endowed with ACE inhibitory activity have also been investigated recently, via in silico studies, for their ability to prevent interaction among the COVID-19 spike glycoproteins and the host cell dipeptidyl peptidase-4 (DPP-4, [34]); however, these promising results are still lacking validation by in vitro or in vivo studies.

Recently, we investigated ACE inhibitory activity by peptide sequences, derived from the enzymatic hydrolysis of whey proteins and predicted by molecular docking and related prioritization studies. This approach allowed us to obtain three peptides—MHI, IAEK, and IPAVF—with ACE inhibitory IC_50_ values equal to or lower than 25 μM [35]. Importantly, IPAVF and the related one-residue-longer sequence IPAVFK also exhibited DPP-4 inhibitory activity and antimicrobial activity against Gram-positive bacteria [33,34].

Building on these observations, we explored the potential of our recently studied ACE inhibitory peptides (i.e., MHI, IAEK, and IPAVF) in interfering with SARS-CoV2 3CL^pro^ functioning. With this in mind, we carried out an integrated theoretical and experimental investigation, involving first, molecular docking studies to rationally evaluate the putative chance of binding and then, in vitro testing for validation. Due to the occurrence of coinfection with a common virus (influenza viruses, human metapneumovirus, and seasonal coronaviruses) in SARS-CoV-2 positive specimens [36,37], peptides were also assayed for their ability to inhibit human rhinovirus 3C protease (EC: 3.4.22.28) in order to evaluate the peptides’ exploitation as broad-spectrum antiviral agents.

## 2. Materials and Methods

### 2.1. Molecular Docking

SARS-CoV-2’s main protease crystal structure was fetched from the Protein Data Bank by retrieving the entry 7L0D [38]. The protein was treated with the Protein Preparation Tool [39,40] available from Schrödinger Suite (New York, NY, USA). Such a method allows for optimizing the crystal structure by removing water molecules, adjusting the side-chain conformation, and adding the missing hydrogen atoms. The peptides to be docked were processed by employing the Ligprep Tool (Schrödinger, New York, NY, USA, for more information, see [41]) to generate all possible tautomers, as well as the protonation states at the physiological pH. The grid-box was generated so as to be suitable for standard precision (SP) peptide-docking protocol and, thus, was centered on the center of mass of the cognate ligand. Satisfactorily, Glide software (Schrödinger, New York, NY, USA) [42] could properly replicate the original binding pose of the co-crystallized molecule, returning a root mean square deviation (RMSD) value as small as 1.025 Å.

The induced-fit docking protocol [43,44] was employed, using Glide with an OPLS3e force field [45] to analyze the binding mode of the selected ligands, together with conformational changes within the receptor. Such changes are not allowed in standard docking protocols. In detail, side-chain conformation predictions were performed on residues within 6 Å from the ligand poses, together with the Glide SP redocking of each protein–ligand complex structure within 30.0 kcal/mol from the lowest energy. 

The molecular mechanics/generalized born surface area (MM-GBSA, accessed on January 2022 [46]) method was employed in the last stage of the study for the computation of the binding free energies (ΔG) between the proteins and ligands. The Prime package (Schrödinger, LLC, New York, NY, USA) [47], available in the Schrödinger 2020-4 suite (New York, NY, USA), was used for this purpose. 

For completeness, -additional molecular docking analyses were carried out against the HRV 3C protease by retrieving entry 2XYA [48] from the Protein Data Bank (https://www.rcsb.org, accessed on 10 January 2022) and are included in the Supplementary Information.

### 2.2. In Vitro Screening of the Antiviral Activity

Synthetic peptides were purchased (purity > 95%; GenScript, Leiden, The Netherlands) and resuspended in MQ water, then assayed at the concentrations of 2, 1, 1, 0.6 0.2 and 0.02 mg/mL (corresponding to 500 to 5 μM for MHI, from 420 to 4.2 μM for IPAVF, and from 366 to 3.6 μM for IAEK). In the case of peptides with a value of relative inhibition (RI) percentage that was higher than 40–50% at the lowest assayed concentration, further dilutions were made. 

#### 2.2.1. SARS-CoV-2 3CL Protease Assay

The in vitro screening of enzyme inhibition activities was evaluated by using 3CL Protease, untagged (SARS-CoV-2 Assay Kit, Catalog #: 78042-1, BPS Bioscience, Inc., Allentown, PA, USA). According to the manufacturer’s protocol, a fluorescent substrate, SARS-CoV-2 3CL Protease (GenBank Accession No. YP_009725301, amino acids 1-306 (full-length), expressed in an *Escherichia coli* expression system, MW = 34 kDa) and a buffer composed of 20 mM Tris, 100 mM NaCl, 1 mM EDTA, and 1 mM DTT, pH 7.3, was used for the inhibition assay. The protease inhibitor, GC376 MW 507.5 Da, with an IC_50_ value of 0.017 μM was used as the positive control. Initially, 30 μL of diluted SARS-CoV-2 3CL protease, at the final concentration of 15 ng, was pipetted into a 96-well plate containing pre-pipetted 10 μL quantities of each test compound (final concentrations of each peptide ranged from 2 to 0.0002 mg/mL in the wells). The mixture was incubated at room temperature for 30 min with slow shaking. Afterward, the reaction was started by adding the substrate (10 μL), dissolved in the reaction buffer to 50 μL final volume, at a concentration of 40 μM, then the plates were incubated for 4 h at room temperature with slow shaking. The plates were sealed. Fluorescence intensity (Fi) was measured with the Varioskan microtiter plate-reader (Varioskan Flash, Thermo Fisher, Milan, Italy), exciting at a wavelength of 360 nm and detecting at a wavelength of 460 nm. Each sample was assayed in triplicate.

The percentage of relative inhibitions (*RI* %) was calculated as follows:

$$\%\;Relative\;Inhibition{\left(\begin{array}{c} \%\;\\ RI \end{array}\right)}^{}=\left(\frac{FiC-FiT}{FiC}\right)\times 100$$

where *C* is the control and *T* is the test peptide or inhibitor.

#### 2.2.2. HRV 3C Protease Determination Assay

The inhibition activity of synthetic peptides was assayed against human rhinovirus 3C protease (HRV 3C^pro^; EC:3.4.22.28), a cysteine protease that recognizes the cleavage site of Leu-Glu-Val-Leu-Phe-Gln*Gly-Pro. Protease activity in the presence or absence of peptide inhibitors was determined colorimetrically, as reported, with an HRV 3C Protease Inhibitor Screening Kit (Catalog #: ab211089, Abcam, Cambridge, UK) according to the manufacturer’s instructions. The kit also included a protease inhibitor as a positive control.

Briefly, the screening sample compound wells contained 10 μL of the test compounds at each concentration (final concentration/well from 1 to 0.05 mg/mL) or 10 μL of inhibitor, or 10 μL of assay buffer in the case of enzyme control, and 50 μL of enzyme solution. After the incubation of the plate at room temperature for 15 min, 40 μL of the substrate solution was added to each reaction, and absorbance (OD = 405 nm) was measured in a kinetic mode for 1–2 h at 37 °C on the Varioskan Flash microplate reader. Each sample was assayed in triplicate. 

The percentage of relative inhibitions (*RI*) was calculated as follows:

$$\%\;Relative\;Inhibition\;{\left(\%RI\right)}^{}=\left(\frac{slopeC-slopeT}{slopeC}\right)\times 100$$

where *C* is the control and *T* is the test peptide or inhibitor.

### 2.3. Statistical Analyses

Results related to the 3CL protease inhibition percentage were subjected to a square root arcsin transformation in order to meet the homogeneity-of-variance assumptions, following Levene’s test. The univariate general linear model (GLM) procedure, carrying out a two-way analysis of variance (ANOVA, *p* < 0.05) using the IBM SPSS Statistics release 20 (IBM, Armonk, NY, USA) to evaluate the main and interaction effects of concentration levels and the assayed peptide types on the inhibition percentage of viral protease. Whenever required, the simple main effects of peptides and the assay control inhibitor on viral protease inhibition percentage were also examined, applying a one-way ANOVA (*p* < 0.05). Multiple comparisons among individual means for each assayed peptide and concentration level were performed using honestly significant difference (HSD)Tukey’s test (*p* < 0.05). A multiple regression test was run to predict the inhibition percentage of 3CL protease activity from different peptide concentrations. 

The half-maximal inhibitory concentration (IC_50_) of peptides on SARS-CoV-2 3CL protease was calculated by using the software package SigmaPlot12 (Systat Software, Inc. SigmaPlot for Windows, San Jose, CA, USA) and referred to the micromolar range.

## 3. Results and Discussion

The chance of targeting SARS-CoV-2 main protease was rationally assessed by employing numerous structure-based approaches [49]. These studies were aimed at predicting the potential of the three small peptides (i.e., IAEK, IPAVF, and MHI) to act as antiviral inhibitors of SARS-CoV-2 main protease, as well as understanding the molecular interactions governing the recognition and the engagement of the binding site [50].

These sequences, endowed with high ACE inhibitor activity, were designed by the molecular pruning of longer β-lactoglobulin-derived peptides (e.g., IIAEKTKIPAVF, and MHIRL); these latter were, in turn, purified and identified after the enzymatic hydrolysis of whey, for its transformation from a by-product of cheese manufacture to a high added-value compound [33]. The assumption that IAEK, IPAVF, and MHI could be good candidates for SARS-CoV-2 3CL^pro^ inhibition was suggested by their sequences, which contain hydrophobic and aromatic amino acids that are able to interact with the hydrophobic regions of the 3CL^pro^ active site, such as the S2 pocket [51]. In addition, bioactive peptides, such as ACE inhibitor ones, usually exhibit multifunctional properties that, once proved, make them excellent candidates for the development of multi-target drugs [33,34]. Similarly, this latter evidence has pushed other authors into investigating their activity as inhibitors of the SARS-CoV-2 main protease [52].

**Figure 1:**
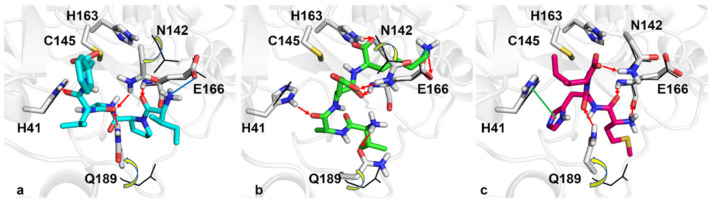
Panels (**a**–**c**) report the best pose returned from docking simulations for IPAVF (cyan sticks), IAEK (green sticks), and MHI (magenta sticks) peptides, respectively. Red arrows and green and blue lines depict hydrogen bonds, π-π, and electrostatic interactions, respectively. Black wireframes show the original side-chain conformation of the 7L0D crystal structures. Yellow arrows highlight the shifting of the side chains from their original positions, due to the induced fit.

We preferred to use the induced fit docking protocol instead of the standard methods, the former being suited to exploring with higher accuracy and reliability the nature and the type of molecular interactions by considering both the ligand and binding site flexibility. In this respect, the effect of the induced fit on the key binding site residues (i.e., H41, N142, C145, H161, E166, and Q189) is assessed by computing the deviations from the crystallographic pose for each protein-oligopeptide complex; the following RMSD values, equal to 1.51 Å, 1.39 Å and 1.24 Å, are measured for IPAVF, IAEK, and MHI, respectively. Interestingly, we observed that Q189 and N142 are more sensitive to the induced fit, as these two residues experienced significant conformational changes, promoting a better fit and the easier accommodation of the three small peptides through the formation of cooperative hydrogen bonds. The N142 χ1 and Q189 χ1 and χ2 torsional angle shifts, due to the induced fit, were reported in Table S1 of the Supplementary Information. Furthermore, the chance of interaction with the catalytic residue H41 and, in addition, with the key residue E166 is supposed to be crucial for the effective inhibition of the target [53,54]. As shown in Figure 1, the three peptides can form a network of hydrogen bonds within the binding pocket. Specifically, IPAVF can interact with the side chains of N142, H41, and Q189, and with the backbone of E166. Furthermore, its protonated nitrogen head can trigger an electrostatic interaction with the negatively charged side-chain of E166. As far as IAEK is concerned, hydrogen bonds with H41, N142, Q189, H163, and E166 were also detected, and the protonated arm of its terminal lysine residue can engage in electrostatic interaction with the side-chain of E166. Regarding MHI, the same hydrogen bonds with E166, N142, and Q189 were observed, and, notably, π−π interactions with H41 through the imidazole ring of its histidine residue were also detected. 

From an energetic point of view, we employed the OPLS3e force field to quantify the docking scores, and the MM-GBSA method to account for the binding of free energies. Interestingly, the values of the docking scores as well as of the binding free energies calculated for the three peptides were much better than those calculated for the reference X-ray-solved cognate ligand, ML188. For the sake of comparison, all the values are reported in Table 1.

In addition, a more detailed analysis of the terms of the binding free energy function indicated that the Coulomb and van der Waal energy contributions were determinants for IPAVF, with values equal to −43.15 kcal/mol and −70.55 kcal/mol, respectively. On the other hand, the strongest hydrogen bonding contribution with a value of −6.13 kcal/mol was for IAEK, due to the presence of two charged side chains within its sequence.

Taken together, all the above-described results concerning the in silico investigations made us confident of the potential antiviral action of these natural small peptides in contrasting SARS-CoV-2 main protease, and this encouraged us to run experimental validations.

Recently, Behzadipour et al. [31] screened several di- and tri-peptides in silico, resulting from the simulated digestion of bovine milk proteins, for their SARS-CoV-2 M^pro^ inhibitory activity using molecular docking. Twenty peptides (with at least one aromatic-hydrophobic amino acid residue at the C-terminal side) showed the best binding energy but this was less than those obtained in the present work. In particular, three among these peptides, originating from the in silico proteolysis of β-caseins, β-lactoglobulin, and αs2-casein, were able to form at least two hydrogen bonds and achieve π-alkyl hydrophobic interactions with the catalytic residues, C145 and H41, of SARS-CoV-2 M^pro^, respectively. Other authors [32,34,55] have reported food-derived peptides with virtual 3CL^pro^ inhibitory activities, showing several potential advantages in terms of high binding affinity, bioavailability, and cost-effective synthesis. 

In the past, a representative group of tripeptide aldehydes (CBZ-Leu-Phe-Gln-CHO) was prepared and analyzed to demonstrate poor inhibitory activity against 3C^pro^. However, peptides that were modified with a primary amide (Gln-γ-CONH2) were more active, revealing carbonyl oxygen–hydrogen bonds with the H161 and Oγ of T142 [56]. Our three small peptides were also subjected to docking simulations within the binding site of 3C^pro^ and similar interactions were experienced, although these yielded more disappointing scoring values. Additional details are available in the Supplementary Information.

Based on the theoretical analyses, the inhibitory activity of the three investigated peptides was experimentally evaluated against SARS-CoV-2 3CL^pro^ and HRV 3C^pro^, both employing the catalytic dyad (His-Cys) for their functionality.

Satisfactory experimental results were observed, following the in vitro inhibition screening of the assayed peptides regarding SARS-CoV-2 3CL^pro^ activity.

The half-maximal inhibitory concentrations (IC_50_) of the three assayed peptides against SARS-CoV-2 3CL^pro^ were measured and are shown in Table 2, confirming IPAVF and IAEK as being more active than MHI; in particular, the IC_50_ value of IPAVF was the best and was comparable with those obtained for the ACE inhibitor peptides against SARS-CoV-2 3CL^pro^, after in silico analyses [52]. The experimental data used to determine IC_50_ are reported in Table S3 in the Supplementary Information. 

Similar inhibitory activities toward SARS-CoV2 in the μM range have been identified for synthetic tetrapeptides, pentapeptides, and octapeptides, as recently reviewed by Heydari et al. [57]. Moreover, the in silico hydrolysis of marine fish proteins by gastrointestinal enzymes released oligopeptides containing 1–3 aliphatic amino acids (A, L, V, I) with a high affinity toward SARS-CoV-2 3CL^pro^; some of these peptides were also predicted to play a role as dual binders toward SARS-CoV-2 3CL^pro^ and monoamine oxidase A [58].

It is noteworthy that no appreciable activity was observed when testing the three small peptides against HRV 3C protease, the RI percentage being lower than a value of ca. 5% at a concentration of 1 mg/mL. A synoptic view is shown in Table 3. This finding is in agreement with our predictive docking studies. Interested readers can look at Figure S1 and Table S2 of the Supplementary Information for more details.

During the COVID-19 pandemic, several variants of SARS-CoV-2 emerged that, nevertheless, showed mutations in the binding domain of the spike protein receptor [59,60]. In all the variants of concern of the virus, no mutation in the M^pro^ gene was recorded [61]. This suggests that any M^pro^ inhibitor could also be effective against multiple variants of the same virus. 

As already demonstrated for Nirmatrelvir [62], it is expected that the inhibitors studied in this work can block viral replication; therefore, they can also reduce infection. Nevertheless, unlike the currently authorized antivirals, the peptides studied could have very few or mild side effects and potentially have broad-spectrum applications. In particular, the peptides studied have been shown to have possible anti-hypertensive effects that would ameliorate the clinical conditions of COVID-19 patients. Finally, biologically active peptides would be cheaper and can be easily transformed into derivatives with improved pharmacological properties.

It is noteworthy that the three proposed peptides represent the minimum active sequences with the desired biological properties toward the SARS-CoV-2 3CL protease. This finding opens the way for the synthesis of peptides with improved activity. Their conversion into therapeutic peptides is, indeed, a further challenging task aimed firstly at tackling two intrinsic stumbling blocks: the low membrane permeability and the poor in vivo stability [63]. Inspired by consolidated medicinal chemistry strategies, backbone and side-chain modifications could be of great help. The former is normally pursued to improve the proteolytic stability of the peptides and includes, for instance, the replacement of L-amino acids with D-amino acids [64], the addition of methyl-amino acids [65], and the inclusion of β-amino acids [66] and peptoids [67]. The latter, instead, aims at exploring changes to improve the binding affinity and selectivity [68]. Together with these design approaches, targeted delivery strategies could also be exploited to overcome the inherent drawbacks of peptides [69]. These approaches could indeed be very valuable to obtain real therapeutic peptides; peptides with protease inhibitory activity have already been studied for various viruses, such as the Dengue virus, West Nile virus, and hepatitis C virus [57].

On the other hand, IAEK, IPAVF, and MHI are naturally included within whey proteins [33,35]; although their purification requires time and cost with a putative low yield, the fractionation of a mixture containing several inhibitory peptides may be an advantageous experimental design step toward the development of nutraceutical supplements with a more efficient manufacturing process and improved activity.

## 4. Conclusions

In this work, the inhibitory activity of the whey-derived bioactive small peptides MHI, IAEK, and IPAVF against viral proteases was evaluated for the first time, indicating their possible role in blocking the replication processes of SARS-CoV-2. 

Molecular docking studies predicted the relevant interactions between the peptides and key amino acid residues of the enzyme catalytic pocket of 3CL^pro^. These results were validated by in vitro experiments that confirmed the highest antiviral activity for IPAVF and IAEK against 3CL^pro^. These peptides of natural origin were previously obtained by the enzymatic hydrolysis of whey proteins and also displayed ACE inhibitory activity. It is noteworthy that their short sequences facilitate their synthesis as well as changes to their structure to improve stability and enhance activity. The results herein open the door to new opportunities for the development of dual-target small peptides that are endowed with antiviral 3CL^pro^ and inhibitory ACE activities.

## Figures and Tables

**Table 1:** Values of the docking score and of MM-GBSA free energy of the best poses obtained through induced-fit docking.

	Docking Score (kcal/mol)	MM-GBSA (kcal/mol)
IPAVF	−10.967	−83.43
IAEK	−10.318	−76.24
MHI	−9.338	−78.80
ML188 cognate ligand	−5.283	−68.03

**Table 2:** Experimental IC_50_ values for the inhibition of SARS-CoV-2 3CL^pro^.

	IC_50_ (*μ*M)	95% Confidence Interval	
MHI	2700.62	1186.17	6145.84
IPAVF	1.21	0.02	9.53
IAEK	154.40	137.18	291.60
GC376 (inhibitor)	0.017	0.05	0.042

**Table 3:** The relative inhibition percentage (RI %) of HRV 3C protease by the assayed small peptides.

Peptides	Concentration for Well	Relative Inhibition (RI %)
MHI	250 μM	4.92 ± 1.02
IPAVF	210 μM	5.30 ± 1.06
IAEK	183 μM	4.54 ± 0.56

## Data Availability

The data presented in this study are available on request from the corresponding author. The data are not publicly available due to privacy issues.

## Supplementary Materials

The following supporting information can be downloaded at: https://www.mdpi.com/article/10.3390/biomedicines10051067/s1, Figure S1: Panels (a), (b), and (c) report the best pose returned from docking simulations for IPAVF (cyan sticks), IAEK (green sticks) and MHI (magenta sticks) peptides, respectively. Red arrows depict hydrogen bonds. Black wireframes show the original side-chain conformation of the 2XYA crystal structures of rhinovirus 3C protease. Table S1: Docking induced-fit variation of the torsional angles χ1 for N142 and of χ1 and χ2 for Q189 from the X-ray structure (PDB_ID: 7L0D) of SARS-CoV-2 3CL^pro^. Table S2: Comparative docking score values for SARS-CoV-2 3CL^pro^ and HRV 3C^pro^. Table S3: Relative inhibition percentage (RI %) of SARS-CoV-2 3CL^pro^ by the three small peptides assayed at different concentrations.
