# Supplementary material for: Rational Discovery of Antiviral Whey Protein-Derived Small Peptides Targeting the SARS-CoV-2 Main Protease
Source: Biomedicines. 2022 May 4;10(5):1067. doi: 10.3390/biomedicines10051067 (PMC9139167; doi:10.3390/biomedicines10051067)
Supplement: Supplementary file 1 [file biomedicines-10-01067-s001.zip › biomedicines-1647395-supplementary.pdf]

Table S1. Docking induced-fit variation of the torsional angles $\chi1$ for N142 and of $\chi1$ and $\chi2$ for Q189 from the X-ray structure (PDB_ID: 7L0D) of SARS-CoV-2 3CL^{pro}.

	N142	Q189	
	$\chi1$ (°)	$\chi1$ (°)	$\chi2$ (°)
7L0D	-70.4	174.4	59.1
IPAVF-IF	-63.2	-168.8	81.9
IAEK-IF	-77.9	-173.1	76.9
MHI-IF	-77	-169	158.1

In the present work, molecular docking simulations were also performed towards HRV3C protease, aiming to help recognize the three oligopeptides as potential dual or selective inhibitors against 3CL and 3C proteases. HRV3C protease crystal structure was retrieved from the Protein Data Bank with entry 2XYA. Protein and ligands were treated and optimized following the same methodology reported in main text. Best docked poses were shown in Figure S1.

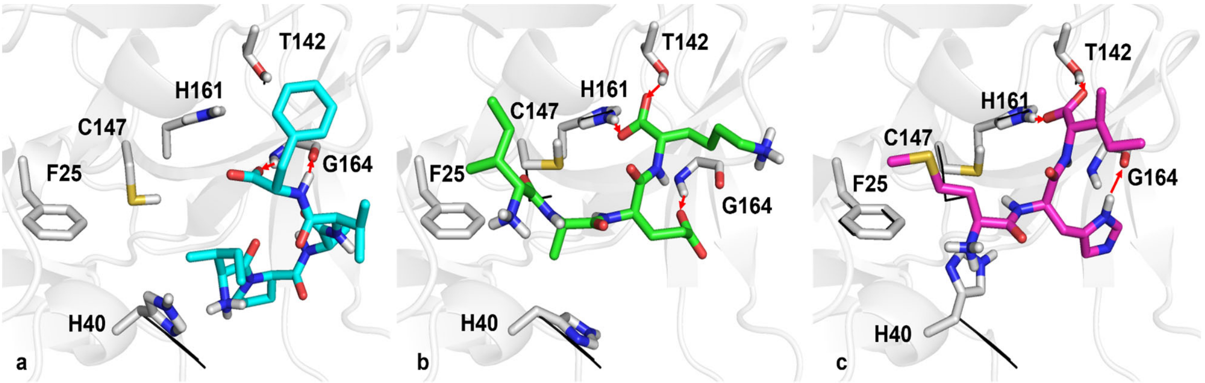

Figure S1. Panels (a), (b) and (c) report the best pose returned from docking simulations for IPAVF (cyan sticks), IAEK (green sticks) and MHI (magenta sticks) peptides, respectively. Red arrows depict hydrogen bonds. Black wireframes show the original side chain conformation of the 2XYA crystal structures of rhinovirus 3C protease.

As observed in Figure S1, IPAVF, IAEK and MHI did show no robust interactions towards the HRV3C protease binding site: G164 is the only residue involved in hydrogen bonds for the three compounds, while H161 and T142 can establish hydrogen bonds only with IAEK and MHI peptides. Interactions with catalytic residue C147 - H41 were not detected.

The weakness of the binding towards HRV 3C protease was also confirmed from an energetically point of view as shown in the following comparative table.

Table S2. Comparative docking score values (kcal/mol): SARS-CoV-2 3CLpro *vs* HRV 3C^{pro}.

	SARS-CoV-2 3CLpro	HRV 3Cpro
IPAVF	-10.967	-3.859
IAEK	-10.318	-6.817
MHI	-9.338	-5.943

Table S3. Relative inhibition percentage (RI %) of SARS-CoV-2 3CL protease by the three small peptides assayed at different concentrations. *

Peptides	Concentration for well (μM)	Relative Inhibition (RI %)
MHI	500	61.54 ± 2.95 (a, C)
	250	49.91 ± 1.74 (b, C)
	150	38.70 ± 3.24 (c, D)
	50	36.81 ± 0.26 (c, D)
	5	17.82 ± 2.34 (d, F)
IPAVF	420	65.02 ± 6.28 (a, B)
	210	70.43 ± 0.76 (a, B)
	0126	57.28 ± 0.38 (b, C)
	42	51.97 ± 4.34 (c, C)
	4.2	50.18 ± 3.39 (c, C)
	0.42	42.92 ± 3.54 (c, D)
	0.042	27.03 ± 2.19 (c, E)
IAEK	366	84.00 ± 3.39 (a, A)
	183	87.69 ± 1.61 (a, A)
	109.8	49.70 ± 3.16 (b, C)
	36.6	46.59 ± 3.79 (c, D)
	3.6	46.45 ± 3.48 (c, D)
	0.36	40.53 ± 1.26 (c, D)
	0.036	34.56 ± 1.13(d, D)
GC376 (inhibitor)	100	92.17 ± 0.83 (A)

*Values are means ± standard deviations. Means with different lowercase and uppercase letters are significantly ($P < 0.05$) different across concentrations and among the peptides, respectively as performed by post hoc Tukey's test.

By analyzing the dose-response relationships of each peptide towards 3CL^{pro} activity, a statistically significant ($F(10, 38) = 19.739$, $p < 0.0001$) concentration-dependent inhibition was found. In particular, with MHI concentration decreasing, its inhibitory activity lowered following a simple power trend ($F(1, 13) = 228.155$, $p < 0.0001$; $R^2 = 0.846$) in reference of which to values less than 250 μM, the inhibitory effect of the peptide dramatically decayed. Relative inhibition percentage by different IAEK doses was best fit to a cubic model ($F(3, 17) = 66.080$, $p < 0.0001$; $R^2 = 0.921$) that partially explained the dichotomic inhibitory effect at high and low peptide concentrations. Conversely, IPAVF behavior towards 3CL^{pro} activity was adequately represented by a log trendline (($F(1, 19) = 118.150$, $p < 0.0001$; $R^2 = 0.861$), suggesting a higher stability of the relative inhibition in relation to peptide amounts in comparison to those of other assayed peptides.
